# Assessing the perspective of well-being of older patients with multiple morbidities by using the LAVA tool - a person-centered approach

**DOI:** 10.1186/s12877-021-02342-3

**Published:** 2021-07-16

**Authors:** B. Wild, V. S. Wurmbach, F. Böhlen, M. K.-P. Kusch, H. M. Seidling, P. Reich, M. Hartmann, W. E. Haefeli, H. C. Friederich, J. Slaets

**Affiliations:** 1 Department of General Internal Medicine and Psychosomatics, Heidelberg University Hospital, 69120 Heidelberg, Germany; 2 Department of Clinical Pharmacology and Pharmacoepidemiology, Heidelberg University Hospital, 69120 Heidelberg, Germany; 3 Cooperation Unit Clinical Pharmacy, Heidelberg University Hospital, 69120 Heidelberg, Germany; 4 Department of Internal Medicine, Univ Medical Center Groningen, University Groningen, Groningen, Netherlands

**Keywords:** Geriatric patients, Patients’ perspective, Patient-centred care, Shared decision making, Normative assessment, Patients’ priorities, Multimorbidity, Patients’ needs

## Abstract

**Background:**

Older patients with multiple morbidities are a particularly vulnerable population that is likely to face complex medical decisions at some time in their lives. A patient-centered medical care fosters the inclusion of the patients’ perspectives, priorities, and complaints into clinical decision making.

**Methods:**

This article presents a short and non-normative assessment tool to capture the priorities and problems of older patients. The so-called LAVA (“Life and Vitality Assessment”) tool was developed for practical use in seniors in the general population and for residents in nursing homes in order to gain more knowledge about the patients themselves as well as to facilitate access to the patients. The LAVA tool conceptualizes well-being from the perspectives of older individuals themselves rather than from the perspectives of outside individuals.

**Results:**

The LAVA tool is graphically presented and the assessment is explained in detail. Exemplarily, the outcomes of the assessments with the LAVA of three multimorbid older patients are presented and discussed. In each case, the assessment pointed out resources as well as at least one problem area, rated as very important by the patients themselves.

**Conclusions:**

The LAVA tool is a short, non-normative, and useful approach that encapsulates the perspectives of well-being of multimorbid patients and gives insights into their resources and problem areas.

**Supplementary Information:**

The online version contains supplementary material available at 10.1186/s12877-021-02342-3.

## Background

Older patients with multiple morbidities are a particularly vulnerable population that is likely to face complex medical decisions at some time in their lives. They differ from patients suffering from only one disease in a number of important aspects: (1) Irreversible disease burden is an emerging part of a patient’s life that in turn modifies individual health goals and pushes curative care into the background; concomitantly, concerns regarding autonomy, living without pain, and overall quality of life move to the foreground. (2) Treatment guidelines rarely consider co-morbidities and thus co-medication can be contraindicated and drug interactions may require dose adjustment [1]. (3) Considering the patient’s medication management capacity, the risk of medication errors multiplies and targeted strategies for error prevention are increasingly important (quaternary prevention). (4) Lastly, the variability in terms of coping strategies, treatment burden, and health goals increases.

For the above reasons, clinicians and researchers in the geriatric field are promoting a major change in health care, namely a more personal approach – an approach that primarily assesses the patient’s needs and priorities and aligns available clinical evidence with these wishes instead of vice versa [2, 3].

The focus of a person-centered approach lies not solely on the cure of chronic illnesses, but rather on the improvement (even optimization) of both the functionality and quality of life. In a person-centered approach, health care providers try to incorporate the patient’s perspectives [4, 5]. Person-centered care emphasizes the limitations of a disease-centered approach and asks for the evaluation of the needs, values, and preferences of patients [6]. For many years, person-centeredness has been advocated as an important dimension of the quality of health care.

To date, many tools are available that help assess a patient’s preferences, but more often than not they are in regard to specific clinical decisions (i.e. treatment yes/no). A recent review regarding preferences in old age pharmacotherapy reported that out of 55 identified instruments the majority of these were designed to assess a patient’s preferences in a disease-specific context while only three questionnaires accounted for multimorbidity [7]. Furthermore, only a limited number of these tools focus on prioritization of health outcomes [8]. Unfortunately, these tools often apply distinct choices, consequently leaving the patient with predefined options and lack a comprehensive assessment of the patient’s preferences as relevant to their own life. In general, all these assessment instruments are either questionnaire-based [9] or (lengthy) qualitative interviews with patients.

An interesting biopsychosocial approach to the assessment of patients’ needs and preferences is given by the Camberwell Assessment of Need for the Elderly (CANE) [10]. The CANE includes 24 items related to the needs of older patients plus two additional items for care givers. Using the CANE questionnaire older people – as well as their caregivers – can indicate whether a need is met or unmet in their life [11]. However, the CANE focuses on the needs of older people with a mental illness and is thus not easily transferrable to the somatic setting of multimorbid patients.

Recently, Blaum et al. [12] described the development and feasibility testing of a process to describe the priorities of patients with multiple conditions in clinical settings. This identification process involves (1) the primary clinician inviting the patient to participate, and (2) trained facilitators who explore the values and preferences of the patient in an interview. This approach also underscores the assumption that an assessment of older patients with multiple conditions by applying only questionnaires or interviews with normative questions does not necessarily lead to a deeper understanding of the patient’s situation and priorities – primarily because a normative assessment method directs and limits the information flow and the genuine narrative of a patient. However, a downside of the identification process as presented by Blaum et al. [12] are the prerequisites of trained facilitators and the considerable amount of time needed that may not be available in a standard clinical setting.

In this article, we present an assessment tool that is able to capture the priorities and problems of older patients that is based on a rather playful and non-normative approach. The so-called LAVA (“Life and Vitality Assessment”) tool was developed in 2016 by J. Lindenberg and J. Slaets of the Leyden Academy on Vitality and Ageing. It was developed for practical use for seniors in the general population and for residents in nursing homes in order to gain more knowledge about the patients themselves (which, in turn, could be used in health care decisions) as well as to facilitate access to the patients. The LAVA tool conceptualizes well-being from the perspectives of older individuals themselves rather than from the perspective of an outsider, i.e., the perspective of the professional and/or researcher. The main aim of the instrument is to support older people to become aware of their most important sources of well-being and how these sources contribute to their experienced well-being in their current life situation. The LAVA tool is feasible without specific training and expert knowledge and entails the possibility of exploring the patient’s perspective on life in medical settings, in a short amount of time, without directing the narrative process by means of normative questions.

The aim of this article is to present the LAVA tool to a broader readership, explain its application, and demonstrate its benefit with selected patient cases in order to make it public and usable for clinicians and researchers working with middle-aged and older people.

## Methods

The LAVA tool was developed in 2016 by the Leyden Academy on Vitality and Ageing for pragmatic use in population research and for resident and nursing homes. Eight focus groups were held with a total of 54 individuals aged 55–85 to discuss what older people find important in their lives and to identify their sources of well-being. A digital version was used in a population sample of 375 older persons, representative in age, gender, and region for the general Dutch population of 55 years and older.

**Fig. 1:**
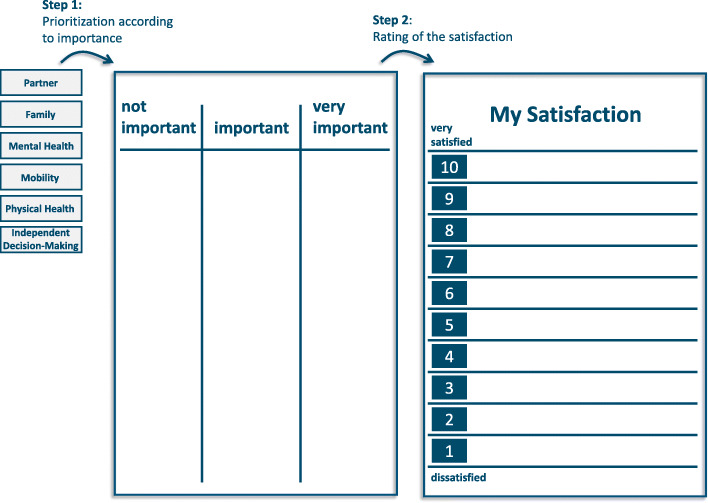
Schematic illustration of the LAVA-Tool

The LAVA tool is applied in two steps. In the first step, participants receive small plastic plates with printed terms on it. In the original LAVA tool, there are 30 plates with expressions in Dutch. The terms – such as ‘physical health’, ‘mobility’, ‘mental health’, ‘independent decision-making’, ‘partner’, and ‘family’ – are drawn from domains such as health, autonomy, life circumstances, social environment, and family. Participants are invited to assign the 30 plates to three different categories classified as ‘not important’, ‘important’, and ‘very important’ for their life and current situation (Fig. 1).

In a second step participants are told to only consider the terms rated as ‘very important’. The other plates are laid aside. Participants then rate their current satisfaction with each term on a board with a scale from 1 to 10 (i.e. 1 = very unsatisfied to 10 = very satisfied) (Fig. 1).

At the end of the assessment, all the terms rated as ‘very important’ lay on the board and are assigned to the different levels of the satisfaction scale. This gives a visual and clear picture of the resources and problems in the life of the patient; it facilitates further exploration by going through the picture and inviting the participants to talk in more detail about their situation.

In 2018, we started to use the LAVA tool in the frame of an ongoing research study (“Prioritization by participation – a method to integrate needs, values, and complaints of older patients with multiple morbidities into treatment planning” (PACT)). The aim of the study is to improve the treatment of older multimorbid patients by including their personal perspective into treatment planning. We translated the terms into German and adapted the 30 plates (reflecting 30 areas of life) to our purpose – this resulted in a total of 25 plates. During the course of this adaptation several of the original terms were removed (e.g. ‘voluntary work’ and ‘luxury goods’) or allocated under new broader terms (‘friends/neighbours’ instead of ‘friends, acquaintances’ and ‘neighbours’). Additionally, in order to focus more on the physical and mental health of the participants in the context of the ongoing study, a few new terms were added (e.g. ‘sleep quality’ and ‘joy of living’). The original 30 terms of the LAVA as well as the 25 terms used in the ongoing PACT study are noted in Supplemental Table 1.

For exploring the use of the LAVA tool in the ongoing PACT study we chose a non-digital approach. The translated and adapted LAVA tool was then integrated into the assessment of multimorbid patients in the frame of the PACT study. In order to become familiar with the patients the assessment started with the INTERMED for the Elderly Interview (IM-E) [13]. Following the IM-E the LAVA tool was applied.

The IM-E is a validated half-structured interview that asks for information regarding the four domains of the patient’s biological, psychological, social, and health care related characteristics**.** The questions in each domain are related to a time axis that is divided into past, present, and future. The answers of the individual questions are scored by means of a four-level rating scale. The total score reflects the amount of health care needs of the participant. A patient with a total score ≥ 21 is considered as having complex health care needs [14].

In the frame of the ongoing PACT study the IM-E and LAVA assessments were conducted with multimorbid older patients by a psychologist, medical assistant doctor, or pharmacist. The patients first completed the INTERMED interview and then applied the LAVA tool. Afterwards, the study assistant explored the most important topics reported by the patients as well as the evaluation of their current satisfaction. During the assessment the participants were asked to talk about the topics and their satisfaction in a personal narrative. Additional data about patients’ diagnoses and medication were collected by a review of the medical notes. The interviews were recorded, then used for a description of the various cases of the participants. For this particular paper, three cases of the study sample were selected to elucidate the possibilities and benefits of the LAVA assessment as it pertains to a medical setting. As we did not specifically ask participants about what they thought would change their ratings the treatment suggestions presented in the discussion of the cases were hypothetically developed based on our knowledge after the assessment was completed.

## Case reports

### Case 1

#### Description

The patient was a male in his early seventies admitted to an integrative ward of a University Hospital, with acute decompensated heart failure. He suffered from severe dyspnea and fatigue; an upgrade of his pacemaker to CRT-D (cardiac resynchronization therapy with defibrillation) was planned. The medical history of the patient was comprised of: chronic heart failure NYHA (New York Heart Association) 3; implantation of dual-chamber pacing with single-chamber defibrillation (dual-chamber-ICD); PTCA (percutaneous transluminal coronary angioplasty) stent; atrial fibrillation; arthritis urica.

In a short interview preceding the assessment with the LAVA the patient reported that his wife had a severe disease and was cared for by him and their children. He said that he was highly concerned about what would happen to his wife if his surgery (upgrade of pacemaker to CRT-D) failed or if he suddenly died of his heart disease. He had previously worked in a company, and was now self-employed. In the past he had been engaged in sports. In his current situation the pursuit of these hobbies was no longer possible because of his physical condition.

**Fig. 2:**
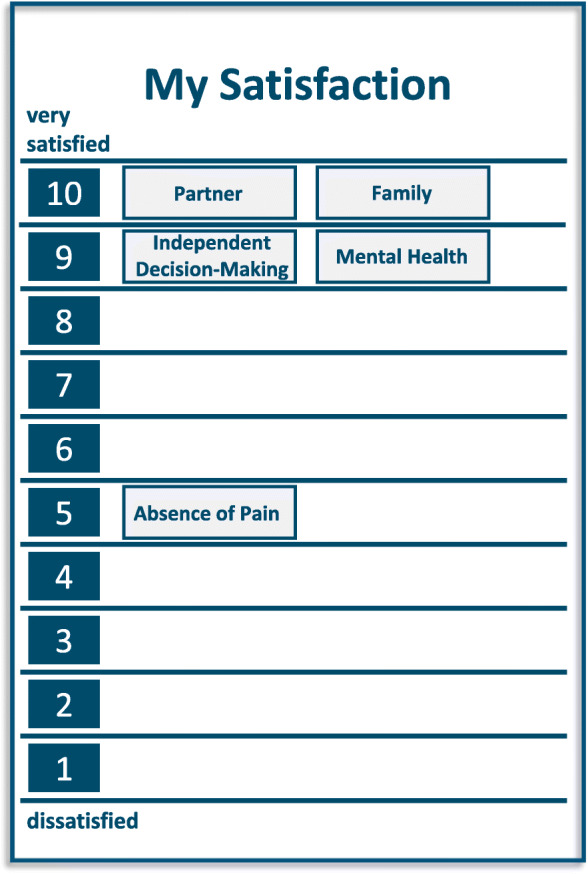
Illustration of the second part of the LAVA-Tool of patient case 1

Figure 2 shows the LAVA self-assessment of the patient.

The LAVA picture is very lucid with five terms categorized by the patient as very important for his life and current situation. Four of the important topics were rated as very satisfying (9–10), interestingly, one of the topics was ‘family’. The family rating does not, however, reflect a husband’s worries about his wife as caregiver – which could be indicative of a fragile family situation.

One of the important aspects, the ‘absence of pain’, was rated as problematic. When the interviewer explored the LAVA picture the patient told her that his whole body was in pain and that he attributed this to his state of fatigue that had already lasted for about 1 year.

#### Discussion

For this patient, the application of the LAVA tool led to very clear information regarding necessary treatment. In the preceding interview (led by specific lead questions that addressed, among other things, physical health and pain) the information about ineffective (or non-targeted) pain management was not clearly described (or captured by the interviewer). It could be that the non-normative approach of the LAVA tool – sorting a number of live aspects by using small plates to indicate satisfaction or dissatisfaction with life – made it easier for the patient to specifically point out this problem area.

When we retrospectively evaluated the appropriateness of the medication we saw that the patient had been diagnosed with arthritis urica and received Allopurinol 100 mg 1–0–0 (paused) to decrease high blood uric acid levels, but no pain medication had been prescribed so far. No other diagnoses were included in his record to explain the high degree of pain he was experiencing. Thus, following the LAVA self-assessment a new evaluation of the pain symptomatology would be recommended including both an orthopaedic and psychological assessment. A new pain medication could be required in combination with physiotherapy or osteopathy.

### Case 2

#### Description

This patient was a female in her late fifties visiting a general practitioner for a sick note due to pain after cervical disc herniation; she had a long history of pain due to fibromyalgia. In a previous interview the patient revealed that her son had died a few years ago of an accident. Since then she had withdrawn from social life and suffered from a sleep disorder. The medical history of the patient was comprised of cervical disc herniation, fibromyalgia, and hypothyreosis.

**Fig. 3:**
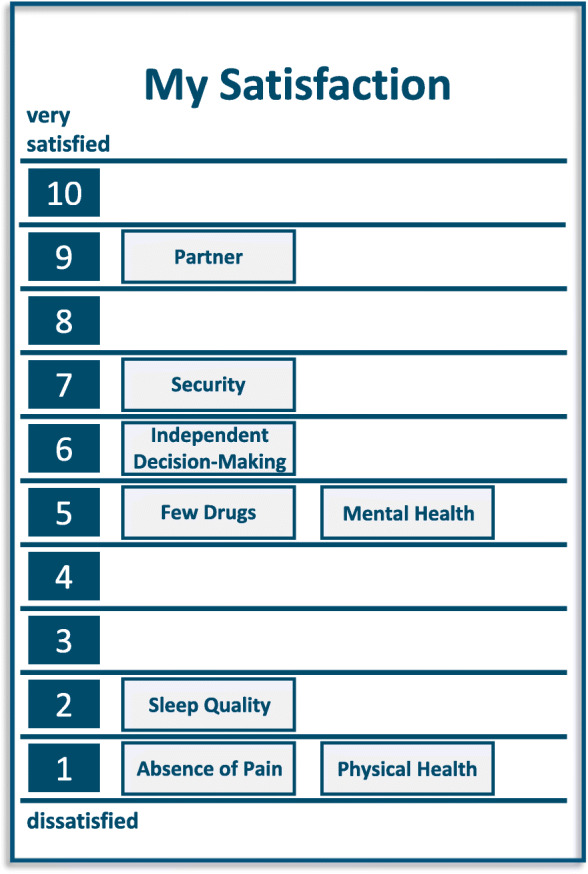
Illustration of the second part of the LAVA-Tool of patient case 2

Figure 3 shows the LAVA self-assessment of the patient.

This patient rated eight terms as very important to her life and current situation. She was very satisfied with her partner and quite satisfied with the number of medications she was taking, mental health, and other aspects of life. However, the aspects of physical health, sleep quality, and absence of pain were rated as very unsatisfying. When exploring these aspects of her life, she confirmed that her pain and reduced physical health were mainly related to cervical disc herniation and fibromyalgia. Regarding the sleep problems the patient admitted that she was sleeping maximally 1 h at a time because she always thought of her dead son.

#### Discussion

For this patient the application of the LAVA tool deepened the discussion about the traumatic life event of losing her son. While the patient had already revealed this major life event and her sleeping problems in the preceding interview, the depth and seriousness of the sleeping problems became manifest only after applying the LAVA tool. We therefore speculated that the rather unconventional way of categorizing and valuing aspects of life triggered the patient’s willingness to talk about the severity of her sleeping disorder and profound mental burden.

Regarding the sleep disorder, the patient had already been diagnosed with fibromyalgia and received Zopiclone 3,75 mg p.r.n. to improve sleep quality and Piroxicam 20 mg p.r.n. (not prescribed by GP) for the reduction of pain. Following the LAVA self-assessment, a new evaluation of the combined pain/sleep symptomatology would be recommended including a psychological interview to assess possible comorbid depression and, additionally, an adaptation of the medication. This could lead to a change in medication (i.e. termination of Zopiclone treatment and a prescription of a sleep-inducing, pain-reducing antidepressant). In addition, we surmised that psychotherapeutic treatment could be useful for the patient to process the loss of her son; also, that the pain medication with Piroxicam should be checked and possibly altered.

### Case 3

#### Description

This patient was a male in his early sixties. He was admitted to an integrative ward of a University Hospital, with a progressing two-vessel coronary heart disease. The patient was scheduled for bypass surgery. The medical history of the patient comprised a Non-ST-elevation myocardial infarction. A percutaneous transluminal coronary angioplasty and a stent implantation had been conducted in the past.

In a short interview preceding the LAVA assessment the patient reported that he had noticed a worsening of his coronary disease under physical exertion in his daily life before hospitalization. Nevertheless, the extent of deterioration that was diagnosed during his hospitalization, surprised him and he described an uncertainty because he did not know what might have caused the worsening of his disease nor how to positively influence the progression of the disease (e.g., life style modifications).

**Fig. 4:**
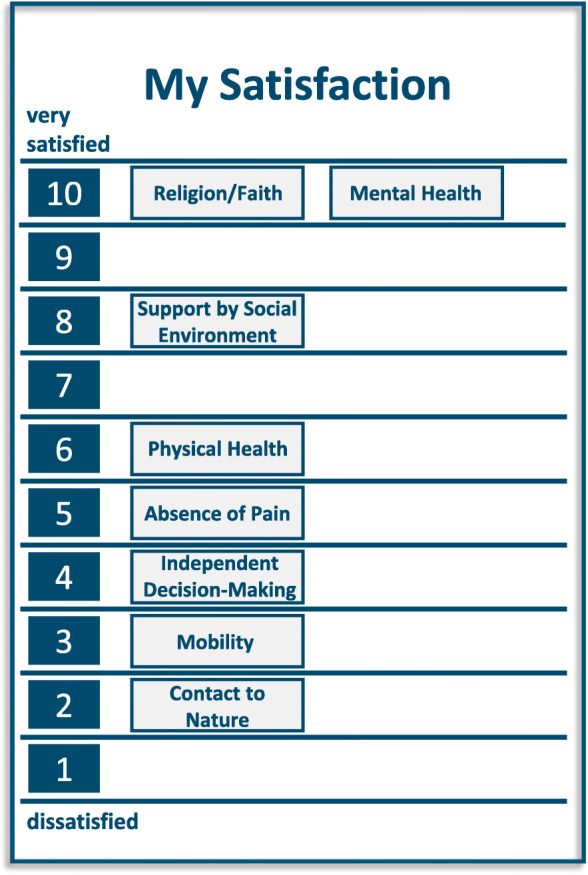
Illustration of the second part of the LAVA-Tool of patient case 3

Figure 4 shows the LAVA self-assessment of the patient.

When using the LAVA-Tool the patient rated eight terms as very important for his life. The three topics the patient was most dissatisfied with were mobility, contact to nature, and the ability to make decisions independently. The negative rating of mobility and contact with nature were probably influenced by the patient’s current situation in the hospital and by his disease. However, his dissatisfaction with his ability to make decisions independently could be attributed to his lack of knowledge concerning his disease and the expected improvement of his condition and mobility after the bypass surgery. A patient consultation that focused on a shared decision and understanding of the link between the treatment aims and his current negative experiences in life could therefore be helpful.

#### Discussion

For this patient, the application of the LAVA tool led to the conclusion that the patient had a need for information. In this case, more information about the disease and the planned surgical intervention could probably help the patient to reduce his uncertainty and to feel more empowered to actively participate in his treatment. In the preceding interview the importance of the information need was not fully captured by the interviewer. The LAVA tool – forcing the patient to weigh his satisfaction with important aspects in his life – made it easier to capture his priorities, thereby showing a potential to improve his situation with a simple measure (i.e. patient information). One could argue that more information and a better understanding of the planned intervention would not have changed the somatic state of the patient. However, and most importantly, for patients with multiple conditions an improvement in well-being and a reduction of fear and uncertainty could make an important difference to their quality of life.

## Discussion

The LAVA tool is an assessment instrument that can be used to assess the perspectives, priorities, and main problems of middle-aged and older people with multiple conditions [15]. The focus is on what matters to the patient in his or her present life and what is therefore relevant for actual treatment decisions. The assessment takes only 5–10 min, can be applied in various settings, and is easily applicable for persons from different health care sectors.

By using a non-normative approach, the assessment is not driven by the attitude or questions of an interviewer but rather by the older persons themselves. This aspect constitutes an important difference compared to other methods. By using normative questions – whether using questionnaires or interviews – interviewers usually lead the persons into predefined areas. Even when using open questions, it can happen that an interviewer misses the main resources and problems of a patient just because it is not the topic of the question. It is true that the LAVA tool also only encompasses a limited number of aspects of life. It is therefore possible that specific areas of resources or problems are missed by the LAVA assessment. However, we had the impression that the presented aspects of life are rated very openly and sincerely by older patients – more openly in a self-directed manner than in a comprehensive interview.

The two-step approach of the LAVA tool to assess the priorities and central problems of the patients is particularly interesting: First, a person has to balance the various aspects of life and determine the most important ones. Second, the person indicates to what extent the most important aspects of life are satisfactory in his/her life. The balancing and indicating is done by using the visual, verbal, and tactile dimension. Selected aspects of life are weighed, resulting in a picture that, based on the structure and content, tells much about the life of the person. Interestingly, this non-normative approach of the LAVA tool appears to be like a ‘door opener’ for older people to point out their most problematic areas of life.

The three cases presented here were chosen to elucidate the usefulness of the LAVA tool, even when a longer interview had previously been performed. The three cases were specifically selected to demonstrate three main problems of older persons with multiple conditions. Pain and sleep disturbances are very frequently experienced by older patients [16, 17] presenting a challenge for health caretakers to find appropriate treatment. The interesting outcome of the LAVA assessment was that the multimorbid patients had the confidence to clearly point out their problems and to refer to possible relationships between symptoms and life experiences. Of course, there will be patients where the LAVA tool will not disclose more than already known. However, in these cases one would get a clear picture of resources and problems of the older patient in just 5–10 min. In addition, in many cases just the structure of the final pattern – where the most important aspects of life are laid out on the satisfaction scale – gives some important information about the patient.

The LAVA tool applies quite well to the assessment of complex patients. Complex patients are characterized by high bio-psycho-social health care needs as well as a need for integrated care. Here, case complexity ‘refers to the characteristics that describe how patients with similar types and stages of disease vary in their health care needs and utilization’ [18]. A complex patient presents the healthcare professionals with a variety of problems on different dimensions. The assessment with the IM-E of health care needs in different domains and represented by scores is very different from the narrative LAVA tool assessment. Both approaches have different goals and can be used together. In a person-centered approach we have to combine two different worlds: the normative and disease oriented medical knowledge and the personal narrative context of the patient. There are many tools for the first objective but the second one needs more attention and is difficult to achieve by health care professionals in a medical context. The application of the LAVA tool could help to prioritize problems and associated decisions from the point of view what matters most to the patient.

The LAVA tool also shows several limitations or critical aspects. (1) The self-assessment depends very much on the current situation of the participants. (2) The terms representing the various aspects of life are pre-defined. It is thus possible that an important resource or problem area is not identified by the assessment. (3) The given terms may not apply to all settings and situations. It could therefore be necessary to change or adapt the terms for a specific setting – which again makes it difficult to compare settings and study samples. (4) To date, there is no algorithm to quantify the LAVA assessment. Quantitative comparisons between studies or study samples are therefore not possible.

However, as a slightly philosophical aspect of our discussion we would lastly like to point out that in health care we usually use master narratives in order to formulate the diagnosis of a patient. Words such as ‘dementia’, ‘pain’, ‘heart failure’, and ‘fibromyalgia’ are used for diagnostic classifications. The origin of this classification language in health care was not to improve treatment but to improve teaching and research [19] Over the last two centuries, however, the words of the patients have disappeared from the language of the health care professionals. Master narratives will have different meanings for individual patients and for health care providers and are both limiting and damaging in capturing the narrative identity of a patient [20]. The LAVA tool can help establish a reconstructive narrative to retell the story in a way that recovers the important features of a person’s identity related to their current state. Person-centered care is not possible without reintroducing the narrative of patients – and this is even more mandatory in complex patients. The interesting part of an assessment with the LAVA tool is not in the final words or scoring but in the narratives explaining the meaning of those words. Narrative identity and medical normative diagnoses are complementary in person-centered care and feasible to achieve even in complex patients.

## Conclusions

The LAVA tool is a non-normative approach to identify resources and problem areas of multimorbid patients. This two-step assessment encourages the patient to focus on the aspects of life that are particular important for him or her and to balance them against each other in terms of his or her satisfaction with them. Hence, the LAVA is useful to identify the priorities and needs of the individual patient – especially in the context of multimorbid, complex patients – and, thus promoting shared decision making. Further research is needed to evaluate the implementation of the LAVA in routine care.

## Supplementary Information

**Additional file 1: Table S1.** LAVA domains used in the PACT study.

## Data Availability

Not applicable.

## Abbreviations

LAVA: Life and Vitality Assessment
