# Supplementary material for: Assessing the perspective of well-being of older patients with multiple morbidities by using the LAVA tool - a person-centered approach
Source: BMC Geriatr. 2021 Jul 16;21:427. doi: 10.1186/s12877-021-02342-3 (PMC8285789; doi:10.1186/s12877-021-02342-3)
Supplement: Supplementary file 1 — Additional file 1: Table S1. LAVA domains used in the PACT study. [file 12877_2021_2342_MOESM1_ESM.docx]

**Assessing the perspective of well-being of older patients with multiple morbidities by using the LAVA tool - a person-centered approach**

B. Wild^1^, V. S. Wurmbach^2,3^, F. Böhlen^1^, M. K.-P. Kusch^2,3^, H. M. Seidling^2,3^, P. Reich^1^, M. Hartmann^1^, W. E. Haefeli^2,3^, H. C. Friederich^1^ and J. Slaets^4^

^1^ Department of General Internal Medicine and Psychosomatics, Heidelberg University Hospital, 69120 Heidelberg, Germany

^2^ Department of Clinical Pharmacology and Pharmacoepidemiology, Heidelberg University Hospital, 69120 Heidelberg, Germany

^3^ Cooperation Unit Clinical Pharmacy, Heidelberg University Hospital, 69120 Heidelberg, Germany

^4^ Department of Internal Medicine, Univ Medical Center Groningen, University Groningen, Groningen, Netherlands

**Supplemental Table 1: LAVA domains used in the PACT study**

| **Original terms (translated from Dutch to German)** | **Original terms (English translation)** | **Terms used in the PACT study (German)** | **Terms used in the PACT study (English translation)** |
| --- | --- | --- | --- |
| Bezahlte Arbeit | Occupation | Berufstätigkeit | Occupation |
| Nachbarn | Neighbours | Freunde/Nachbarn | Friends/Neighbours |
| Familie | Family | Familie | Family |
| Finanzen | Finances | Finanzielle Sicherheit | Financial Security |
| Geistige Gesundheit | Mental Health | Geistige Gesundheit | Mental Health |
| Hobbies | Hobbies | Hobbies | Hobbies |
| Haustiere | Pets |  |  |
| Internet und Telefon | Internet and Phone | Internet und/oder Telefon | Internet and/or Phone |
| Kinder | Children |  |  |
| Enkelkinder | Grandchildren |  |  |
| Körperliche Gesundheit | Physical Health | Körperliche Gesundheit | Physical Health |
| Luxusgüter | Luxury Goods |  |  |
| Pflege durch das soziale Umfeld | Care through Social Environment | Unterstützung durch soziales Umfeld | Support by Social Environment |
| Umweltfreundlichkeit | Environmental Friendliness |  |  |
| Mobilität | Mobility | Mobilität | Mobility |
| Natur | Nature | Kontakt zur Natur | Contact to Nature |
| Öffentliche oder private Transportmittel | Public or Private Transportation | Öffentliche oder private Transportmittel | Public or Private Transportation |
| Partner | Partner | Partner | Partner |
| Religion/Glauben | Religion/Faith | Religion/Glaube | Religion/Faith |
| Aussehen | Appearance | Aussehen | Appearance |
| Ausgehen | Going out |  |  |
| Urlaub | Vacation |  |  |
| Sicherheit | Security | Sicherheit | Security |
| Vereine | Clubs |  |  |
| Einrichtungen in der Nachbarschaft | Neighbourhood Facilities |  |  |
| Freunde, Bekanntschaften | Friends, Acquaintances |  |  |
| Freiwilligenarbeit | Voluntary Work |  |  |
| Wohnen | Living | Wohnsituation | Living Situation |
| Selbstständigkeit | Autonomy |  |  |
| Selbst Entscheidungen treffen | Independent Decision-Making | Selbstständige Entscheidungen treffen | Independent Decision-Making |
|  |  | Lebensfreude | Joy of Living |
|  |  | Gewicht | Body Weight |
|  |  | Schlafqualität | Sleep Quality |
|  |  | Möglichst geringes Risiko | Lowest Possible Risk |
|  |  | Schmerzfreiheit | Absence of Pain |
|  |  | Wenig Medikamente | Few Drugs |
|  |  | Freude am Essen | Enjoying Meals |
